# Diagnosis of Leptospirosis: Comparison between Microscopic Agglutination Test, IgM-ELISA and IgM Rapid Immunochromatography Test

**DOI:** 10.1371/journal.pone.0129236

**Published:** 2015-06-18

**Authors:** Roshan Niloofa, Narmada Fernando, Nipun Lakshitha de Silva, Lilani Karunanayake, Hasith Wickramasinghe, Nandana Dikmadugoda, Gayani Premawansa, Rajitha Wickramasinghe, H. Janaka de Silva, Sunil Premawansa, Senaka Rajapakse, Shiroma Handunnetti

**Affiliations:** 1 Institute of Biochemistry, Molecular Biology and Biotechnology, University of Colombo, Colombo, Sri Lanka; 2 Tropical Medicine Research Unit, Faculty of Medicine, University of Colombo, Colombo, Sri Lanka; 3 National Reference Laboratory for *Leptospira*, Medical Research Institute, Colombo, Sri Lanka; 4 Base Hospital Homagama, Homagama, Sri Lanka; 5 Colombo North Teaching Hospital, Ragama, Sri Lanka; 6 Faculty of Medicine, University of Kelaniya, Ragama, Sri Lanka; 7 Faculty of Science, University of Colombo, Colombo, Sri Lanka; Federal University of Pelotas, BRAZIL

## Abstract

**Background:**

Leptospirosis is diagnosed on clinical grounds, and confirmed by microscopic agglutination test (MAT). IgM-ELISA (Serion-Virion) and immunochromatography test (Leptocheck-WB) are two immunodiagnostic assays for leptospirosis. Their sensitivity, specificity and applicability in Sri Lanka have not been systematically evaluated.

**Methods:**

Clinically diagnosed leptospirosis patients (n = 919) were recruited from three hospitals in the Western Province of Sri Lanka, during June 2012 to December 2013. MAT, IgM-ELISA and Leptocheck-WB were performed on all patient sera. MAT titer of ≥400 in single sample, four-fold rise or seroconversion ≥100 in paired samples were considered as positive for MAT. For diagnostic confirmation, MAT was performed during both acute and convalescent phases. Anti-leptospiral IgM ≥20 IU/ml and appearance of a band in the test window were considered as positive for IgM-ELISA and Leptocheck-WB test respectively. Patients with an alternative diagnosis (n = 31) were excluded. Data analysis was performed using two methods, i) considering MAT as reference standard and ii) using Bayesian latent class model analysis (BLCM) which considers each test as imperfect.

**Results:**

MAT, IgM-ELISA and Leptocheck-WB positivity were 39.8%, 45.8% and 38.7% respectively during the acute phase. Acute-phase MAT had specificity and sensitivity of 95.7% and 55.3% respectively, when compared to overall MAT positivity. IgM-ELISA and Leptocheck-WB had similar diagnostic sensitivity when compared with acute-phase MAT as the gold standard, although IgM-ELISA showed higher specificity (84.5%) than Leptocheck-WB (73.3%). BLCM analysis showed that IgM-ELISA and Leptocheck-WB had similar sensitivities (86.0% and 87.4%), while acute-phase MAT had the lowest sensitivity (77.4%). However, acute-phase MAT had high specificity (97.6%), while IgM-ELISA and Leptocheck-WB showed similar but lower specificity (84.5% and 82.9%).

**Conclusions:**

Both IgM-ELISA and Leptocheck-WB shows similar sensitivities and specificities. IgM-ELISA may be superior to MAT during the acute phase and suitable for early diagnosis of leptospirosis. Leptocheck-WB is suitable as a rapid immunodiagnostic screening test for resource limited settings.

## Introduction

Leptospirosis is a globally widespread zoonosis caused by pathogenic spirochetes belonging to the genus *Leptospira*[1]. An estimated 500,000 cases occur annually, with fatality range rising up to 70% in different cohorts[2]. Leptospirosis is endemic to Sri Lanka, with outbreaks occurring every four to five years. A large outbreak took place in 2008, with 7406 reported cases and 204 deaths, giving an incidence rate of 35.7 per 100,000 populations, and case fatality rate of 2.75%[3].

Human hosts commonly acquire infection through skin abrasions and mucosal surfaces following contact with water or soil contaminated with urine of infected rodents or other mammals. Leptospirosis has a wide range of clinical manifestations, from a simple febrile illness to a severe and potentially fatal illness characterized by acute kidney injury, liver derangement, pulmonary haemorrhage, bleeding, and cardiac involvement. In most clinical settings, there is limited availability of specific diagnostic tests, and treating physicians often rely on clinical features to make a probable diagnosis of leptospirosis. This is indeed a problem in areas of high incidence of other infections with similar clinical picture, such as dengue, rickettsial infection, malaria and hantavirus infections[4].

Laboratory diagnosis of leptospirosis is based on several methods: the microscopic agglutination test (MAT), detection of organism DNA by polymerase chain reaction (PCR), isolation of the organism through culture methods, or detection of antibodies to the organism[5]. Isolation of *Leptospira* spp. from clinical samples has low diagnostic sensitivity, requires specialized expertise, and most importantly takes too long to be of use to the treating team[6]. Antigens can be detected by histological, histochemical or immunestaining techniques and *Leptospira* DNA by PCR. Unfortunately, none of these tests are currently suitable for routine laboratory use, because of technical limitations and low sensitivity[5]. MAT is considered the reference immunological test, and detects both immunoglobulin M (IgM) and immunoglobulin G (IgG) class agglutinating antibodies. However, this test requires a high level of technical expertise, and the maintenance of a large panel of live pathogenic *Leptospira* standard cultures. The use of live *Leptospira* organisms also creates a risk of laboratory acquired infection to the laboratory technicians[7]. MAT also gives large number of false negative results in the early course of infection, as IgM antibodies detectable by MAT appear after day 8 of the illness, reach the peak by the fourth week, and furthermore, detectable titers of serovar specific functional antibodies may persist for several months[8–10]. MAT requires testing paired sera collected at appropriate time intervals for an accurate interpretation of results. Thus, while it is of value for epidemiological purposes, there are limitations in its value in the acute clinical setting. Currently, MAT is routinely available only in a central reference laboratory in Sri Lanka, i.e., the National Reference Laboratory for *Leptospira*, Medical Research Institute (MRI), Colombo[11]. At the time of conducting this study, only *Leptospira biflexa* serovar Patoc strain Patoc I was used by the MRI.

There is thus a clear need for reliable and valid rapid diagnostic tests for leptospirosis which can be made available to clinicians, in order to diagnose and treat leptospirosis during early course of infection. The ideal diagnostic test for leptospirosis should have high sensitivity and specificity during the acute phase, be widely available at reasonable cost, and give quick results. Several other immunodiagnostics have been evaluated as alternatives to MAT, such as Ig M detectable enzyme linked immune sorbent assay (IgM-ELISA), dot ELISA, indirect hemagglutination assay (IHA), immunofluorescence assay (IFA), *Leptospira* dipstick test and *Leptospira* immunochromatography test[12–14]. While these are relatively easier to perform when compared with MAT, their diagnostic accuracies have not been fully established. IgM-ELISA shows promise as an alternative to MAT, as many laboratories in tropical countries have facilities to perform the test[15, 16]. Some studies have reported that IgM-ELISA has high sensitivity and specificity[15, 17]. However, one study has been reported from Sri Lanka evaluating a commercially available immunodiagnostic ELISA (InstitutVirion\Serion GmbH, Warburg, Germany) kit showing very low sensitivity and specificity[18]. In this study, the acute phase IgM-ELISA was compared with diagnostic confirmation based on a four-fold rise in titer between acute and convalescent samples, and not against the immunological reference standard MAT. Leptocheck-WB test is a commercially available immunochromatographic test which identifies IgM, does not require any specialized laboratory facilities, and provides results within 15 minutes[13]. Leptocheck-WB has been evaluated in limited studies.

Although MAT is usually considered the immunological ‘gold’ standard for diagnosis, as mentioned above, MAT has inherent flaws. There has been much debate about the validity of using MAT as an immunological gold standard for evaluation of rapid diagnostics[19]. Bayesian latent class modelling, a statistical model which assumes that all tests are imperfect, has been suggested as a more suitable method for evaluating diagnostic tests, including immunodiagnostics for leptospirosis[19–21].

In this study, we evaluated two commercially available tests detecting *L*. *biflexa* serovar Patoc strain Patoc I specific IgM antibodies, and MAT detecting both agglutinating IgM and IgG antibodies against only *L*. *biflexa* serovar Patoc strain Patoc I. We analyzed our findings using two statistical models, i.e., taking MAT as the gold standard, and Bayesian latent class modelling.

## Methods

The Standards for the Reporting of Diagnostic Accuracy Testing (STARD) were adhered to in this study (S1 Checklist)[22].

### Study population

A total of 919 patients were enrolled in this study, from three hospitals in the Western Province of Sri Lanka. The Western Province is the most highly populated province in the country, with a square area of 3709 km^2^ and population of 5.72 million[23]. An analysis of hospital based sentinel surveillance data of leptospirosis over 4 years in Sri Lanka has confirmed that of nearly 4000 suspected cases, 47% were from this province[24]. The three Hospitals were the National Hospital of Sri Lanka (NHSL), Colombo North Teaching Hospital (CNTH) and Base Hospital Homagama (BHH). Patients were recruited from June 2012 to May 2014. Patients over the age of 12 years, with a suspected diagnosis of Leptospirosis, admitted to the medical wards of these hospitals were enrolled. A suspected diagnosis of leptospirosis was defined based on the WHO-LERG epidemiological criteria[25], i.e., acute febrile illness with headache, myalgia, arthralgia, conjuctival suffusion, meningeal irritation, anuria, oliguria, protreinuria, jaundice, hemorrhages, cardiac arrhythmia or skin rash, or a contact history of exposure to water or soil contaminated with urine of infected animals. Patients with a definitive alternative diagnosis on presentation, such as dengue, pneumonia, meningitis, or other bacterial sepsis, and pregnant women were excluded from the study. Data was collected by investigators who were not directly involved in patient care. Demographic and clinical data and laboratory and other investigation findings were collected until the point of discharge or death, using a structured interviewer administered questionnaire.

### Laboratory Methods

Five milliliters of blood were collected by sterile venepuncture and allowed to clot at 37°C, and serum was separated by centrifugation at 800 *g* for 10 minutes. Leptocheck-WB and MAT were performed immediately after recruitment. Sera were stored at -20°C until the performance of IgM-ELISA. All enrolled patients who survived were requested to return for convalescent sampling on day 21 from disease onset, during which 2 mL of blood taken for convalescent MAT.

#### Microscopic agglutination test

MAT was performed at the Reference Laboratory for Leptospirosis, Medical Research Institute, Colombo employing standard procedure[26]. Live organisms of *L*.*bilfexa* serovar Patoc strain Patoc I were cultured and maintained in EMJH (Ellinghausen- McCullough-Jonson-Harris) liquid media at room temperature. Serially diluted from the dilution of 1:100, serum specimens were added to the live *Leptospira* cell suspension in 96well round bottomed microtiter plates, and incubated for two hours at 37°C. Agglutination was examined under a magnification of 20X using dark field microscopy. The reciprocal of the highest dilution agglutinating at least 50% of the *Leptospira* organisms, was considered as the reporting titer. Single acute MAT positivity was defined as a titer of ≥400. Final MAT positivity was defined as a titer of ≥400 in single sample, sero-conversion from negative to a titer ≥100 or a four-fold rise in titer in paired (acute and convalescent) samples[25, 27].

#### Immunochromatography test

Leptocheck-WB (Zephyr Biomedicals, India) test was performed according to manufacturer’s instructions[28]with a small modification. Five drops of running buffer were added following the addition of 20 μL serum to the test window. Although the manufacturer’s instructions state that 10 μL of serum should be added, we performed a preliminary study with a small number of samples using both 10 μL and 20 μL of serum which demonstrated that the positive bands were persistent with 20 μL of serum without altering the actual result. Results were read visually after 15 minutes of incubation at room temperature. Anti-human IgM colloidal gold conjugate forms a complex with IgM antibodies in the sample while it flows through the membrane assembly of the test device. Antigens from *L*. *biflexa* serovar Patoc strain Patoc I are coated on the window 'T' of membrane capture, and immobilize the antibody-conjugate complex if present in the sample. This forms a red color band at the test region 'T'. The un-reacted conjugate and the unbound complex, if any, along with rabbit globulin colloidal gold conjugate move further on the membrane and are subsequently immobilized by the anti-rabbit antiserum coated at the control region 'C' of the membrane assembly, forming a red color band. Presence of bands in the test and control windows was read as positive, while absence of a band in the test window with the presence of control band was read as negative. Absence of a band in the control window was read as invalid test and test was repeated.

#### IgM-ELISA

IgM-ELISA (InstitutVirion\Serion GmbH, Warburg, Germany) was performed according to manufacturer’s instructions[29]. Briefly, rheumatoid factor (RF) absorbent was diluted 1:4 in dilution buffer to obtain RF dilution buffer. This ELISA uses crude antigens from an isolated, concentrated and partially purified extract of *L*. *biflexa* serovar Patoc strain Patoc I, which contains genus specific epitopes for all *Leptospira* serovars. Sera sample was diluted 1:100 in RF dilution buffer and incubated for 15 minutes at room temperature. This is performed for the removal of IgM rheumatoid factors. Standards and diluted samples were transferred to the microtiter wells and incubated at 37°C for 60 minutes in a moist chamber. Residual serum was removed from the wells by washing four times with the wash buffer; anti-human IgM conjugated to alkaline phosphatase was added and incubated at 37°C for 30 minutes in a moist chamber. Wells were washed four times with the wash buffer; substrate *p*-nitrophenyl phosphate was added and incubated at 37°C for 30 minutes in a moist chamber. Sodium hydroxide was added and the enzyme substrate reaction was stopped for the readings. Optical density against the substrate blank was read at 405 nm and at a background of wavelength of 650 nm. Each kit was performed with a negative control, positive control and cut-off calibrator (standards) in duplicate. Absorbance reading of the above in a test obeying the specifications of the Serion ELISA indicates that the test is valid. Results were obtained using the evaluation table provided along with the kit. Interpretation of results for Serion ELISA classic *Leptospira* IgM was as follows: anti-leptospiral IgM <15 IU/ml gives a negative result suggesting no evidence of a recent infection, 15–20 IU/ml gives a borderline result suggesting that may be a recent infection and ≥20 IU/ml gives a positive result which is interpreted as a recent or current infection.

All sera with a positive result for any of the above tests were tested for hantaviral infection, using a commercially available IgM-ELISA kit (InstitutVirion\SerionGmgH, Warburg, Germany). The assay was performed according to the manufacturer’s instructions[30]. Results were obtained using the evaluation table provided along with the kit. This provided quantities of anti-hantaviral IgM in IU per mL and qualitative results: negative (<10 IU/mL) result suggesting no evidence of recent infection, borderline (10 to 15 IU/mL) result suggesting possible recent infection, and positive (≥15 IU/mL) result suggesting a recent or current infection. Borderline results of both ELISAs were considered as negatives. Hantaviral IgM positives were excluded from the analysis.

### Ethics approval

Ethics approval was obtained from the Ethics Review Committee of the Faculty of Medicine, University of Colombo (EC-12-056). Patients were recruited to the study after obtaining informed written consent from the patient, next of kin or care-takers when patients were severe. Informed written consent was obtained from parents or guardian on behalf of patients aged below 18 years.

### Statistical analyses

Statistical analyses were performed using Statistical Package for the Social Sciences (SPSS) version 17.0. We considered positive MAT under two circumstances: a) MAT during the acute phase of illness, a titer of ≥400 (Acute MAT), and b) either acute MAT, or a four-fold rise in MAT titer between acute and convalescent samples, or seroconversion on MAT to a titer of ≥100 (Final MAT). Patients positive on ‘Final MAT’ were considered true positives for leptospirosis for the purpose of gold standard analysis. First, the diagnostic accuracy of ‘acute MAT’ was evaluated with ‘final MAT’ as gold standard, where data was available. Next, sensitivities, specificities, positive and negative predictive values of Leptocheck-WB and IgM-ELISA were calculated with the ‘final MAT’ as the gold standard. Finally we compared both ‘Acute MAT’ and ‘Final MAT’ separately with IgM-ELISA and Leptocheck-WB using Bayesian latent class modelling. The MICE tool (Modelling for Infectious Disease Centre, Mahidol-Oxford Research Unit)[31, 32] was used to perform Bayesian latent class modelling.

## Results

**Fig 1:**
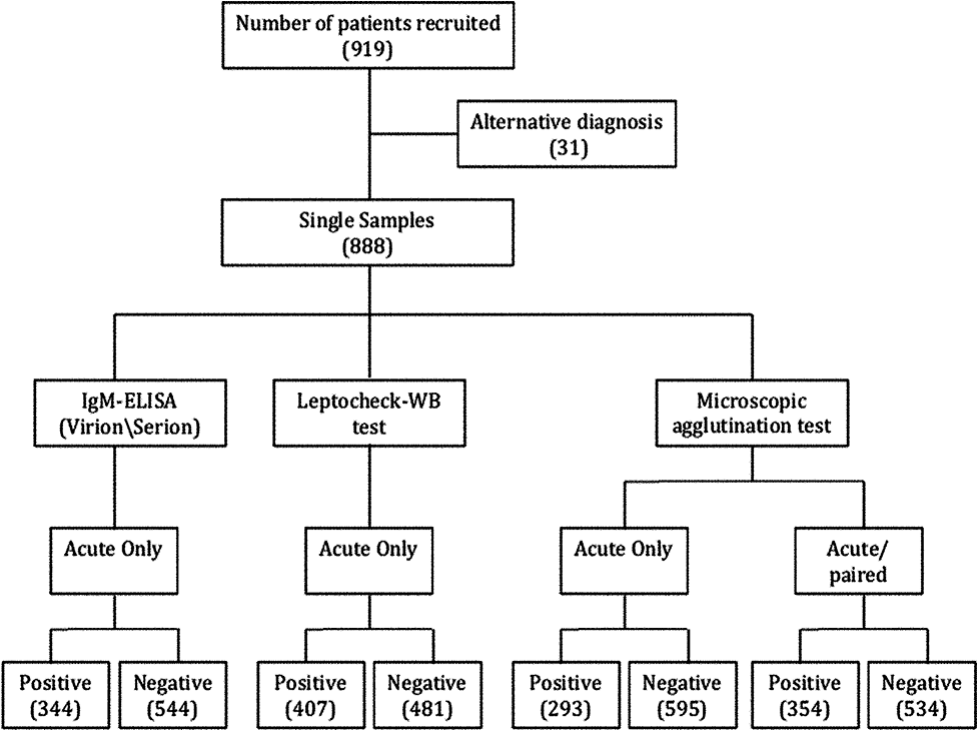
Flowchart showing the participants and the results of leptospirosis diagnostic tests microscopic agglutination test (MAT), Leptocheck-WB and IgM-ELISA.

We enrolled a total of 919 patients with acute fever and a suspected diagnosis of leptospirosis (NHSL-689, BHH -165, CNTH -34). Of these, 31 patients were excluded from the analysis as they were diagnosed as having dengue, typhoid fever, and sepsis or hantaviral infection. Data of 888 patients were included in the final analysis. The male to female ratio was 9:1. Mean age was 42 years (SD±16). Samples were collected at median of 6 days (SD±3.58) after the onset of symptoms. Follow-up samples were received from 255/888 patients. The baseline characteristics of the patients are shown in Table 1. Further details about participants and diagnostic assays are shown in Fig 1.

**Table 1:** Baseline demographic and clinical profile of enrolled patients.

Characteristic		Baseline data
Age, Mean ±SD; (Range)		41.7 ±15.6; (13–80)
Male: Female Ratio		9:1
Exposure to contaminated water		
	Yes	597
	No	256
Occupation		
	Farming	119
	Other	614
	Unemployed	120
Fever		888
Headach		760
Myalgia		778
Nausea and vomiting		459
Conjuctival suffusion		416
Jaundice		196
Acute kidney injury		304
Hemorrhage		225
Lung involvement		12
ICU admissions		35
Received haemodialysis		139
Deaths		26

### Positivity based on MAT

Based on the criteria considered as MAT positivity (i.e., either titer of ≥400 in single sample, or seroconversion from negative to a titer ≥100, or a four-fold rise in titer in paired samples), a total of 354 (39.8%) patients were MAT positive, out of the total of 888 patients included in the final analysis. Of these, 293 patients had a single MAT positive, and another 61 patients were positive based on paired MAT.

### Accuracy of single acute MAT

Using the subset of patients who had both acute and convalescent samples analyzed (n = 255), we compared the accuracy of a single MAT performed during the acute phase of illness (defined as **Acute MAT**), against **Final MAT** (i.e., positivity or negativity based on any of the three MAT criteria). In this cohort, 93 were MAT positive in the acute phase, and 161 were positive for when convalescent samples were considered (Table 2). Acute MAT had a sensitivity of 55.3%, specificity of 95.7%, a positive predictive value (PPV) of 0.95 and a negative predictive value (NPV) of 0.55. While MAT is a highly specific test, it lacks sensitivity during the acute stage of infection.

**Table 2:** MAT during the acute phase compared with overall MAT positivity.

MAT Test	Positive	Negative	Sensitivity (%)	Specificity (%)
**Acute MAT**	93	162	55.3	95.7
**Final MAT**	161	94	100	100

Acute MAT defined as MAT performed on acute serum sample. Final MAT defined as positivity or negativity based on acute MAT, acute and convalescent samples, or seroconversion, and used as the reference standard. Positive and negative values are given as absolute numbers.

### IgM-ELISA and Leptocheck-WB compared with MAT positivity as gold standard

Using a single acute MAT (Acute MAT) as a reference standard, 33% of patients in the cohort had confirmed leptospirosis. Leptocheck-WB had a sensitivity of 84.6% while IgM-ELISA had a sensitivity of 86.0% (Table 3) (S1 and S2 Tables); there was no significant difference in sensitivity between the 2 methods. The specificity of IgM-ELISA [84.5% (81.3%-87.3%)] was significantly higher than that of Leptocheck-WB [73.3% (69.5%-76.8%)]. When a combination of acute samples and paired samples for MAT (i.e., Final MAT) were considered, the proportion of confirmed leptospirosis increased to 43.4% (39.5%-47.5%). There was a significant reduction in the sensitivity of leptocheck-WB test. However, IgM-ELISA retained good levels of sensitivity or specificity.

**Table 3:** Prevalence, sensitivities, specificities and positive and negative predictive values of Leptocheck-WB and IgM-ELISA using the MAT as gold standard and Bayesian latent class models.

	MAT as gold standard (%)* ^ ^		Bayesian latent class model (%)^+^	
Parameters	Acute only	Acute or Paired	Acute only	Acute or Paired
Prevalence	33.0(29.9–36.2)	43.4 (39.5–47.5)	40.8 (37.0–44.9)	43.4 (39.5–47.5)
**MAT**				
Sensitivity	100	100	77.4 (71.8–82.3)	85.4 (80.6–89.6)
Specificity	100	100	97.6 (95.3–99.2)	94.3 (91.2–96.8)
PPV	100	100	95.6 (91.7–98.6)	92.0 (87.6–95.7)
NPV	100	100	86.2 (82.0–89.6)	89.4 (85.3–92.6)
**Leptocheck-WB Test**				
Sensitivity	84.6 (79.9–88.5)	80.8 (76.2–84.7)	87.4 (83.0–91.3)	86.2 (81.5–90.0)
Specificity	73.3 (69.5–76.8)	76.9 (73.0–80.4)	82.9 (79.1–86.1)	84.3 (80.3–87.7)
PPV	60.9 (56.0–65.7)	70.3 (65.5–74.6)	77.8 (72.9–82.4)	80.8 (75.8–85.1)
NPV	90.6 (87.6–93.0)	85.6 (82.0–88.5)	90.5 (86.6–93.5)	88.8 (84.6–92.2)
**IgM-ELISA (Virion\Serion)**				
Sensitivity	86.0 (81.4–89.7)	80.2 (75.6–84.2)	86.0 (81.4–89.7)	86.9 (82.2–91.0)
Specificity	84.5 (81.3–87.3)	88.5 (85.4–91.1)	84.5 (81.3–87.3)	97.5 (95.1–99.7)
PPV	73.3 (68.2–77.8)	82.6 (78.0–86.3)	73.3 (68.2–77.8)	96.4 (92.5–99.5)
NPV	92.5 (89.8–94.5)	86.9 (83.7–89.6)	92.5 (89.8–94.5)	90.6 (86.9–93.7)

*Gold standard model assumed that MAT is perfect (100% sensitivity and 100% specificity; all patients with gold standard test positive are diseased and all patients with gold standard test negative are non-diseased). MAT titer ≥ 400 was considered to be positive. Values shown are estimated means with 95% confidence interval.

^+^Bayesian latent class model assumed that all tests evaluated are imperfect. Values shown are estimated median with 95% credible interval.

### Bayesian latent class modelling for MAT, IgM-ELISA and Leptocheck-WB test

Based on the proportion of patients diagnosed with leptospirosis among this group of patients being 0.41 (0.37–0.45), and using only acute samples (i.e., acute MAT), sensitivities of MAT, Leptocheck-WB and IgM-ELISA were 77.4% (71.8%-82.3%), 87.4% (83.0%-91.3%) and 86.0% (81.4%-89.7%), respectively, and specificities were 97.6% (95.6%-99.2%), 82.9% (79.1%-86.1%) and 84.5% (81.3%-87.3%), respectively.

The proportion of patients diagnosed with leptospirosis among this group of patients using both acute and paired samples was 0.43 (0.39–0.47). Sensitivities of MAT, Leptocheck-WB and IgM-ELISA were 85.4% (80.6%-89.6%), 86.2% (81.5%-90.0%) and 86.9% (82.2%-90.0%) respectively; the specificities were 94.3% (91.2%-96.8%), 84.3% (80.3%-87.7%) and 97.5% (95.1%-99.7%) respectively.

## Discussion

Early and definitive diagnosis of leptospirosis is important to guide the clinician to commence appropriate treatment, and prioritize resource allocation for management of complications. Although MAT is generally considered the immunological gold standard, our analysis shows that MAT has poor sensitivity when performed early; the use of both acute and convalescent samples increases the sensitivity of MAT as a test to diagnose leptospirosis. Bayesian latent class modelling also demonstrated that the sensitivity of MAT was relatively low, but increased when considering both acute and convalescent samples. Historically, MAT is used as the reference standard for the serological assays and widely used for the confirmation of the disease. However, our study suggests that MAT is an imperfect gold standard for the early detection of leptospirosis. MAT detects agglutinating antibodies of both IgM and IgG classes. These functional antibodies take 1–2 days longer than the appearance of *Leptospira* genus specific IgM antibodies. The period for which IgM and IgG antibodies detected by MAT persist following acute infection is a subject of controversy. Infection with certain types of serovars, have been shown to produce longer lasting immunity, such as the Autumnalis serogroup[10]. Nonetheless our study showed high specificity with acute MAT. HoweThus, MAT is useful as a confirmatory test, and for epidemiological purposes.

In our study, the Patoc-1 genus specific strain was used in all three tests (MAT, Leptocheck-WB and IgM-ELISA) that were evaluated. As discussed elsewhere, genus specific antibodies appear earlier than serovar specific antibodies. So at the acute stage of infection, genus specific tests, especially IgM detecting assays are expected to give positive results while serovar specific tests are still not able to detect the antibodies.

The gold standard analysis of our study was compared with the other studies (Table 4). In previous studies, Serion IgM-ELISA’s sensitivity ranges from 48% to 100% and specificity ranges from 88.6% to 98%. Leptocheck-WB test’s sensitivity ranges from 78 to 93.81% and specificity ranges from 86.81 to 98%. These results show a correlation with the results of our present study.

**Table 4:** Results of the study in comparison with other studies.

	Reference	Sample size	Sensitivity	Specificity
IgM-ELISA	Panwala et al [13]	130	93.8	90.1
(Virion\Serion)	Kucerova et al [33]	45	100.0	88.6
	Effler et al [34]	344	48.0	98.0
	Present study	888	86.9	97.5
Leptocheck WB test	T Panwala[13]	130	93.8	86.8
	MG Goris[35]	197	78.0	98.0
	Present study	888	86.1	84.5

High sensitivity and specificity of IgM-ELISA during the acute phase of illness using single sample, make *Leptospira* genus specific IgM detecting ELISA suitable for both early as well as definitive diagnosis. This test also gives high PPV and NPV during the early phase of infection.

Leptocheck-WB also has a high sensitivity and reasonable specificity. It is easy to perform, rapid method that takes only 15–20 minutes, and does not require any special equipment. In comparison, IgM-ELISA has several steps in its procedure, requires a technically skilled person, takes about 4 hours to perform, and requires an ELISA plate reader. Leptocheck-WB test gives consistent results, and the deep color bands, which are stable for more than 12 months. Kit contents are stable and can be transported and stored at ambient temperatures, and are small, portable packages. In our study, the approximate cost per specimen for IgM-ELISA was US $ 3.4 whereas Leptocheck-WB cost was only approximately US$ 1.9. The higher sensitivity and NPV of Leptocheck-WB, together with its lower cost and ease of use, suggests that it would be useful as a screening test. The higher specificity, sensitivity, PPV and NPV of IgM-ELISA suggest that IgM-ELISA is appropriate for confirmation and definitive diagnosis, and may be superior to MAT, especially during the acute phase of illness.

One limitation of our study was the use of *L*. *biflexa* serovar Patoc strain Patoc I as the base for all three diagnostic tests. At the time of conducting this study, this was the only strain for which MAT was available in the reference laboratory in Sri Lanka. Our future studies will incorporate testing against a panel of serovars.

## Conclusion

MAT is an imperfect gold standard serological test for early diagnosis; its high specificity makes it a useful tool for confirmatory diagnosis, however it lacks sensitivity for use in diagnosis of acute illness. MAT would be an important tool for epidemiological purposes, such as identification of infecting serovars, and also to identify the prevalent serovar during an outbreak. IgM-ELISA (InstitutVirion\SerionGmgH, Warburg, Germany) is suitable for early and definitive diagnosis of acute leptospirosis. Leptocheck-WB test is suitable as a screening test for use in resource-limited settings. Our results reiterate the importance of proper evaluation of serological diagnostics[19] using statistical models that assume that all tests are imperfect.

## Supporting Information

S1 Checklist STARD checklist for reporting studies of diagnostic accuracy. (DOC)

S1 Table Results of three diagnostic tests on acute sample (n = 888). (DOCX)

S2 Table Results of three diagnostic tests on acute/paired sample (n = 888) used for MAT. (DOCX)

## References

[1] Bharti AR, Nally JE, Ricaldi JN, Matthias MA, Diaz MM, Lovett MA, et al Leptospirosis: a zoonotic disease of global importance. The Lancet Infectious Diseases. 2003;3(12):757–71. 10.1016/s1473-3099(03)00830-2 14652202

[2] Vijayachari P, Sugunan AP, Shriram AN. Leptospirosis: an emerging global public health problem. Journal of biosciences. 2008;33(4):557–69. . 19208981 10.1007/s12038-008-0074-z

[3] Agampodi SB, Peacock SJ, Thevanesam V, Nugegoda DB, Smythe L, Thaipadungpanit J, et al Leptospirosis outbreak in Sri Lanka in 2008: lessons for assessing the global burden of disease. The American journal of tropical medicine and hygiene. 2011;85(3):471–8. 10.4269/ajtmh.2011.11-0276 21896807 PMC3163869

[4] Izurieta R, Galwankar S, Clem A. Leptospirosis: The "mysterious" mimic. Journal of emergencies, trauma, and shock. 2008;1(1):21–33. 10.4103/0974-2700.40573 19561939 PMC2700559

[5] Musso D LSB. Laboratory diagnosis of leptospirosis: A challenge. Journal of microbiology, immunology, and infection = Wei mian yu gan ran za zhi. 2013;46(4):245–52. 10.1016/j.jmii.2013.03.001 . 23639380

[6] Wuthiekanun V, Chierakul W, Limmathurotsakul D, Smythe LD, Symonds ML, Dohnt MF, et al Optimization of culture of Leptospira from humans with leptospirosis. Journal of clinical microbiology. 2007;45(4):1363–5. 10.1128/JCM.02430-06 17301285 PMC1865830

[7] Sugunan AP, Natarajaseenivasan K, Vijayachari P, Sehgal SC. Percutaneous exposure resulting in laboratory-acquired leptospirosis—a case report. Journal of medical microbiology. 2004;53(Pt 12):1259–62. 10.1099/jmm.0.45735-0 . 15585507

[8] Alder B FS. The antibodies involved in the human immune response to leptospiral infection. Journal of medical microbiology. 1978;11:387–400. 722781 10.1099/00222615-11-4-387

[9] Cumberland P, Everard CO, Levett PN. Assessment of the efficacy of an IgM-elisa and microscopic agglutination test (MAT) in the diagnosis of acute leptospirosis. The American journal of tropical medicine and hygiene. 1999;61(5):731–4. . 10586903 10.4269/ajtmh.1999.61.731

[10] Cumberland P, Everard CO, Wheeler JG, Levett PN. Persistence of anti-leptospiral IgM, IgG and agglutinating antibodies in patients presenting with acute febrile illness in Barbados 1979–1989. European journal of epidemiology. 2001;17(7):601–8. . 12086073 10.1023/a:1015509105668

[11] Agampodi SB, Nugegoda DB, Thevanesam V. Determinants of leptospirosis in Sri Lanka: study protocol. BMC infectious diseases. 2010;10:332 10.1186/1471-2334-10-332 21087520 PMC2994874

[12] Levett PN BS, Whittington CU, Edwards CN, Paxton H. Two methods for rapid serological diagnosis of acute leptospirosis. Clinical and diagnostic laboratory immunology. 2001;8(2):349–51. 10.1128/CDLI.8.2.349-351.2001 11238220 PMC96061

[13] Panwala T MS, Patel P. Seroprevalance of leptospirosis in south gujarat region by eveluating the two rapid commercial diagnostic kits against the MAT test for detection of antibodies to leptospira interrogans. National Journal of Community Medicine. 2011;2(1):64–70.

[14] Sehgal S, Vijayachari P, Sharma S, Sugunan A. Lepto dipstick a rapid and simple method for serodiagnosis of acute leptospirosis. Transactions of the royal society of tropical medicine and hygiene. 1999;93:4 10450439 10.1016/s0035-9203(99)90293-6

[15] Ooteman MC, Vago AR, Koury MC. Evaluation of MAT, IgM ELISA and PCR methods for the diagnosis of human leptospirosis. Journal of microbiological methods. 2006;65(2):247–57. 10.1016/j.mimet.2005.07.015 . 16253361

[16] Bajani MD, Ashford DA, Bragg SL, Woods CW, Aye T, Spiegel RA, et al Evaluation of four commercially available rapid serologic tests for diagnosis of leptospirosis. Journal of clinical microbiology. 2003;41(2):803–9. 12574287 10.1128/JCM.41.2.803-809.2003 PMC149700

[17] Desakorn V, Wuthiekanun V, Thanachartwet V, Sahassananda D, Chierakul W, Apiwattanaporn A, et al Accuracy of a commercial IgM ELISA for the diagnosis of human leptospirosis in Thailand. The American journal of tropical medicine and hygiene. 2012;86(3):524–7. 10.4269/ajtmh.2012.11-0423 22403329 PMC3284374

[18] Reller ME, Bodinayake C, Nagahawatte A, Devasiri V, Kodikara-Arachichi W, Strouse JJ, et al Leptospirosis as frequent cause of acute febrile illness in southern Sri Lanka. Emerging infectious diseases. 2011;17(9):1678–84. 10.3201/eid1709.100915 21888794 PMC3322050

[19] Limmathurotsakul D, Turner EL, Wuthiekanun V, Thaipadungpanit J, Suputtamongkol Y, Chierakul W, et al Fool's gold: Why imperfect reference tests are undermining the evaluation of novel diagnostics: a reevaluation of 5 diagnostic tests for leptospirosis. Clinical infectious diseases: an official publication of the Infectious Diseases Society of America. 2012;55(3):322–31. 10.1093/cid/cis403 22523263 PMC3393707

[20] Speybroeck N, Praet N, Claes F, Van Hong N, Torres K, Mao S, et al True versus apparent malaria infection prevalence: the contribution of a Bayesian approach. PloS one. 2011;6(2):e16705 10.1371/journal.pone.0016705 21364745 PMC3041757

[21] Limmathurotsakul D, Jamsen K, Arayawichanont A, Simpson JA, White LJ, Lee SJ, et al Defining the true sensitivity of culture for the diagnosis of melioidosis using Bayesian latent class models. PloS one. 2010;5(8):e12485 10.1371/journal.pone.0012485 20830194 PMC2932979

[22] Patrick M Bossuyt JBR, David E Bruns, Constantine A Gatsonis, Paul P Glasziou, Les M Irwig JGL, David Moher, Drummond Rennie, Henrica C W de Vet. Towards complete and accurate reporting of studies of diagnostic accuracy: the STARD iniative. British Medical Journal. 2003;326:41–4. 12511463

[23] Western Provincial Council Official Website 2013 [31st October 2014]. Available from: http://www.wpc.gov.lk.

[24] Gamage CD, Amarasekera J, Palihawadana P, Samaraweera S, Mendis D, Janakan N, et al Analysis of hospital-based sentinel surveillance data on leptospirosis in Sri Lanka, 2005–2008. Japanese journal of infectious diseases. 2012;65(2):157–61. . 22446124

[25] Organization WH. Report of the Second Meeting of the Leptospirosis Burden Epidemiology Reference Group. 2011.

[26] John R Cole CRSa ARP. Improved microtechnique for the Leptospiral microscopic agglutination test. Applied Microbiology. 1973;25(6):976–80. 4736794 10.1128/am.25.6.976-980.1973 PMC380950

[27] India WHOCof. Leptospirosis Laboratory Manual. 2007.

[28] Rapid immunochromatographic test for IgM antibodies to Leptospirosis in human serum, plasma and whole blood Instructions Zephyr Biomedicals; 2013 [13th October 2013]. Available from: http://www.tulipgroup.com.

[29] Virion/Serion. Serion ELISA classic Leptospira IgM (quantitatitive) instructions 2013 [7th November 2013]. Available from: http://www.virion-serion.de/fileadmin/templates/tpl1/global/download/flyer/Flyer_ELISA_classic_Leptospira__EN.pdf.

[30] Virion/Serion. Serion ELISA classic Hanta virus puumula IgM (quantitatitive) instructions 2013 [7th November 2013]. Available from: http://www.virion-serion.de/fileadmin/templates/tpl1/global/download/flyer/Flyer_ELISA_classic_Hantavirus_Puumala__EN.pdf.

[31] Lim C, Wannapinij P, White L, Day NP, Cooper BS, Peacock SJ, et al Using a web-based application to define the accuracy of diagnostic tests when the gold standard is imperfect. PloS one. 2013;8(11):e79489 10.1371/journal.pone.0079489 24265775 PMC3827152

[32] Modelling for Infectious disease CEnter. 2013.

[33] Kucerova P, Cermakova Z, Pliskova L, Valenta Z, Pavlis O, Kubickova P. [Comparison of results of two serological methods for diagnosing leptospirosis—microagglutination test and ELISA]. Klinicka mikrobiologie a infekcni lekarstvi. 2011;17(5):173–8. . 22161754

[34] Effler PV, Bogard AK, Domen HY, Katz AR, Higa HY, Sasaki DM. Evaluation of eight rapid screening tests for acute leptospirosis in Hawaii. Journal of clinical microbiology. 2002;40(4):1464–9. 11923374 10.1128/JCM.40.4.1464-1469.2002 PMC140343

[35] Goris MG, Leeflang MM, Loden M, Wagenaar JF, Klatser PR, Hartskeerl RA, et al Prospective evaluation of three rapid diagnostic tests for diagnosis of human leptospirosis. PLoS neglected tropical diseases. 2013;7(7):e2290 10.1371/journal.pntd.0002290 23875034 PMC3708816

