# Supplementary material for: Diagnosis of Leptospirosis: Comparison between Microscopic Agglutination Test, IgM-ELISA and IgM Rapid Immunochromatography Test
Source: PLoS One. 2015 Jun 18;10(6):e0129236. doi: 10.1371/journal.pone.0129236 (PMC4472754; doi:10.1371/journal.pone.0129236)
Supplement: S2 Table — (DOCX) [file pone.0129236.s003.docx]

| **MAT (Acute/Paired)** | **Leptocheck WB** | **IgM ELISA (Virion-Serion)** | **Number (n=888)** |
| --- | --- | --- | --- |
| Positive | Positive | Positive | 245 |
| Positive | Positive | Negative | 41 |
| Positive | Negative | Positive | 40 |
| Negative | Positive | Positive | 43 |
| Positive | Negative | Negative | 36 |
| Negative | Positive | Negative | 78 |
| Negative | Negative | Positive | 16 |
| Negative | Negative | Negative | 389 |
